# Supplementary material for: Silencing CA1 pyramidal cells output reveals the role of feedback inhibition in hippocampal oscillations
Source: Nat Commun. 2024 Mar 11;15:2190. doi: 10.1038/s41467-024-46478-3 (PMC10928166; doi:10.1038/s41467-024-46478-3)
Supplement: Supplementary file 1 — Supplementary Information [file 41467_2024_46478_MOESM1_ESM.pdf]

Supplementary information

Silencing CA1 pyramidal cells output reveals the role of feedback inhibition in hippocampal oscillations

Chinnakkaruppan Adaikkan[1,✉], Justin Joseph[1,+], Georgios Foustoukos[2,+,#], Jun Wang[3,+], Denis Polygalov[2], Roman Boehringer[2], Steven J Middleton[2], Arthur J.Y, Huang[2], Li-Huei Tsai[3,4], Thomas J McHugh[2,5,✉]

Affiliations:

[1]Centre for Brain Research, Indian Institute of Science, Bengaluru, Karnataka 560012, India.
[2]Laboratory for Circuit and Behavioral Physiology, RIKEN Center for Brain Science, Wakoshi, Saitama 351-0198, Japan.
[3]Department of Brain and Cognitive Sciences, Picower Institute for Learning and Memory, Massachusetts Institute of Technology, Cambridge, MA 02139, USA.
[4]Broad Institute of Harvard and Massachusetts Institute of Technology, Cambridge, MA 02139, USA.
[5]Department of Life Sciences, Graduate School of Arts and Sciences, The University of Tokyo, Tokyo, Japan.
+ These authors contributed equally.
[#] Current address: Department of Fundamental Neurosciences, University of Lausanne, Lausanne, 1005, Switzerland.
[✉]Correspondence: chinna@iisc.ac.in (C.A.), thomas.mchugh@riken.jp (T.J.M.)

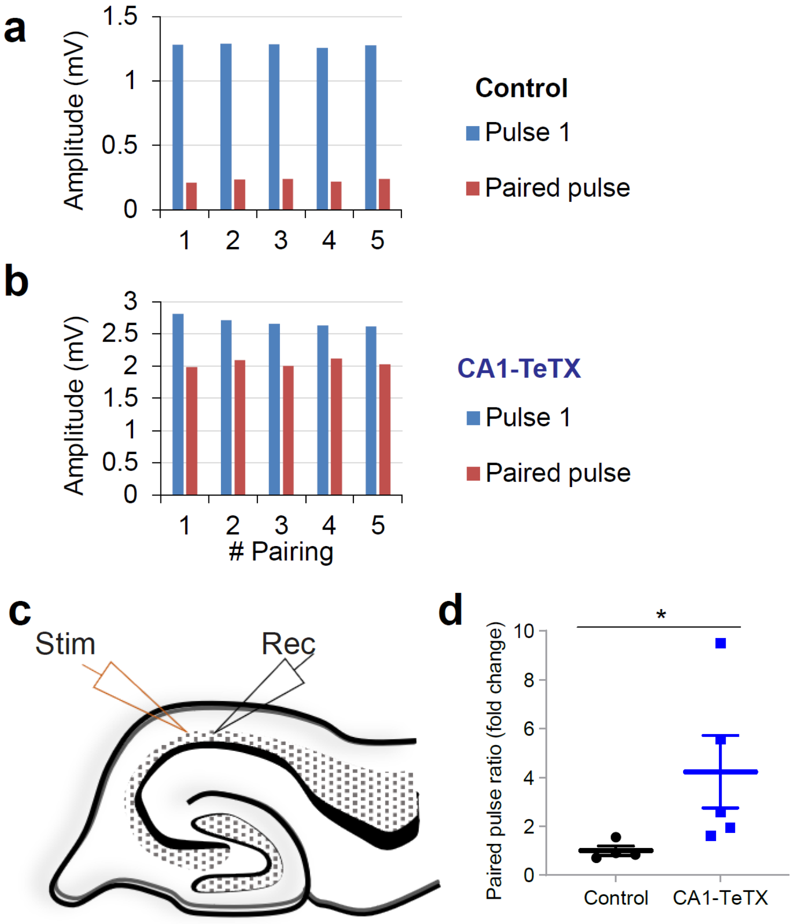

Supplementary Fig. 1: Reduced feedback inhibition in CA1-TeTX mice.

a,b Five repetitive paired-pulse stimulations with an inter-pairing interval of 100 ms were tested. Representative amplitude responses for the five repetitive paired-pulse stimulations are shown from control (**a**) and CA1-TeTX (**b**) mice.

c In a complementary experiment to optogenetics evoked paired-pulse suppression (Fig 1n-1q), we performed electrical stimulation to examine paired-pulse suppression. We stimulated CA1 input and recorded population spikes in CA1.

d Electrically evoked paired-pulse ratio also revealed a reduction in feedback inhibition in CA1-TeTX mice compared to control mice (n = 4 - 5 slices, N = 3 mice/group, Mann Whitney test, P = 0.0159). These findings overall suggest a reduction in feedback inhibition in CA1 in CA1-TeTX mice. Data represents mean ± s.e.m. * indicates P < 0.05, ns= not significant.

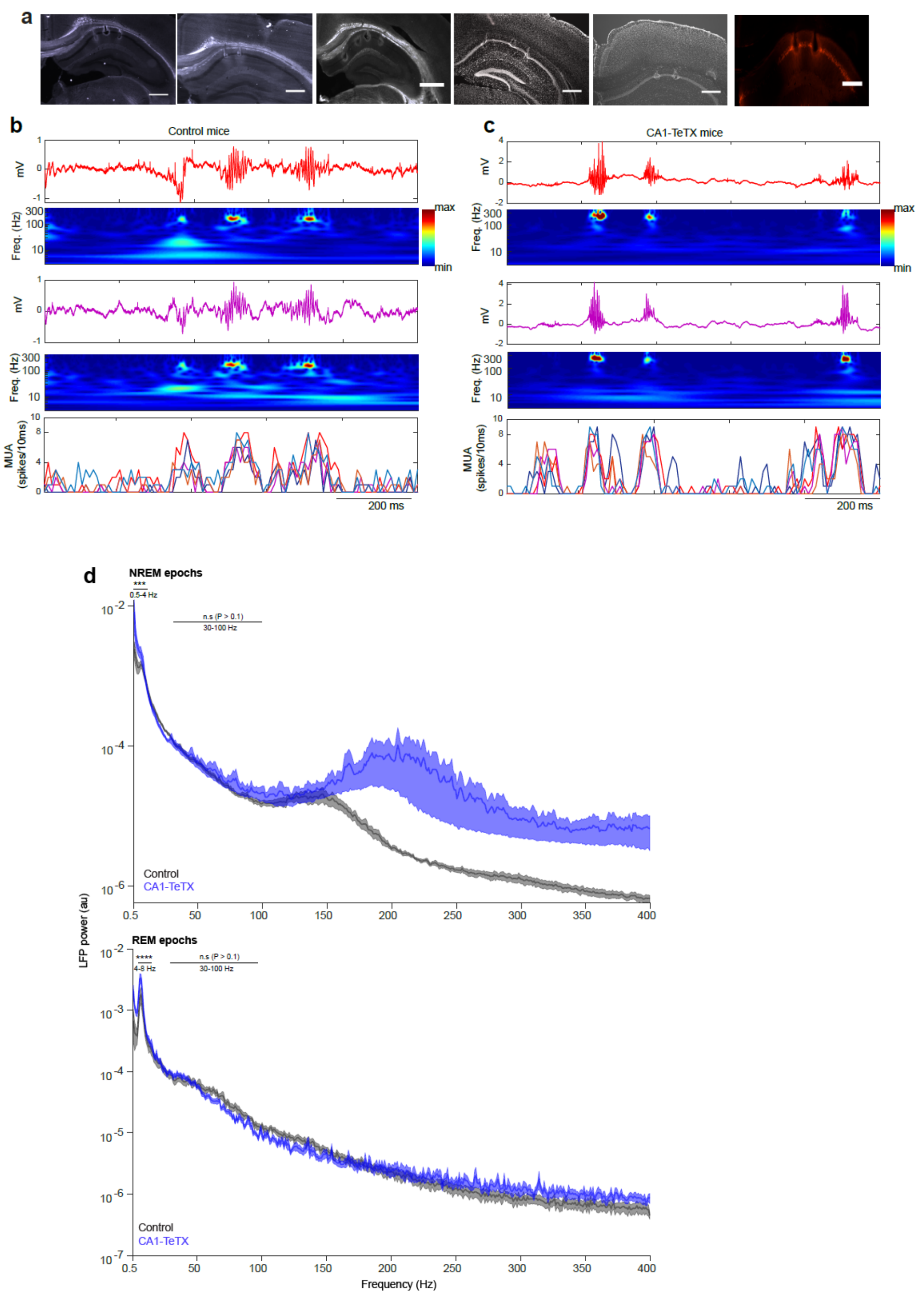

Supplementary Fig. 2: Increased multiunit activity in CA1 in CA1-TeTX mice.
a Photomicrographs show the site of recordings verified by electrolytic lesioning.
b, c Two simultaneously recorded LFP channels and the corresponding LFP scalogram in a control (**b**) and a CA1-TeTX mice (**c**). Multiunit activity (MUA) from five individual tetrodes in each mouse.
d LFP PSD during putative NREM (top, 2 Way ANOVA, groups x frequency interaction $F(401, 4010) = 1.53$, $P < 0.0001$) & REM (bottom, $F(401, 4010) = 4.099$, $P < 0.0001$) epochs. Results from post-hoc tests for delta, theta & gamma bands, corrected for multiple comparisons, are shown at the top. N = 5 Control and 7 CA1-TeTX mice. Data represents mean ± s.e.m. *** & **** indicate $P < 0.001$, & $P < 0.0001$, respectively. ns= not significant.

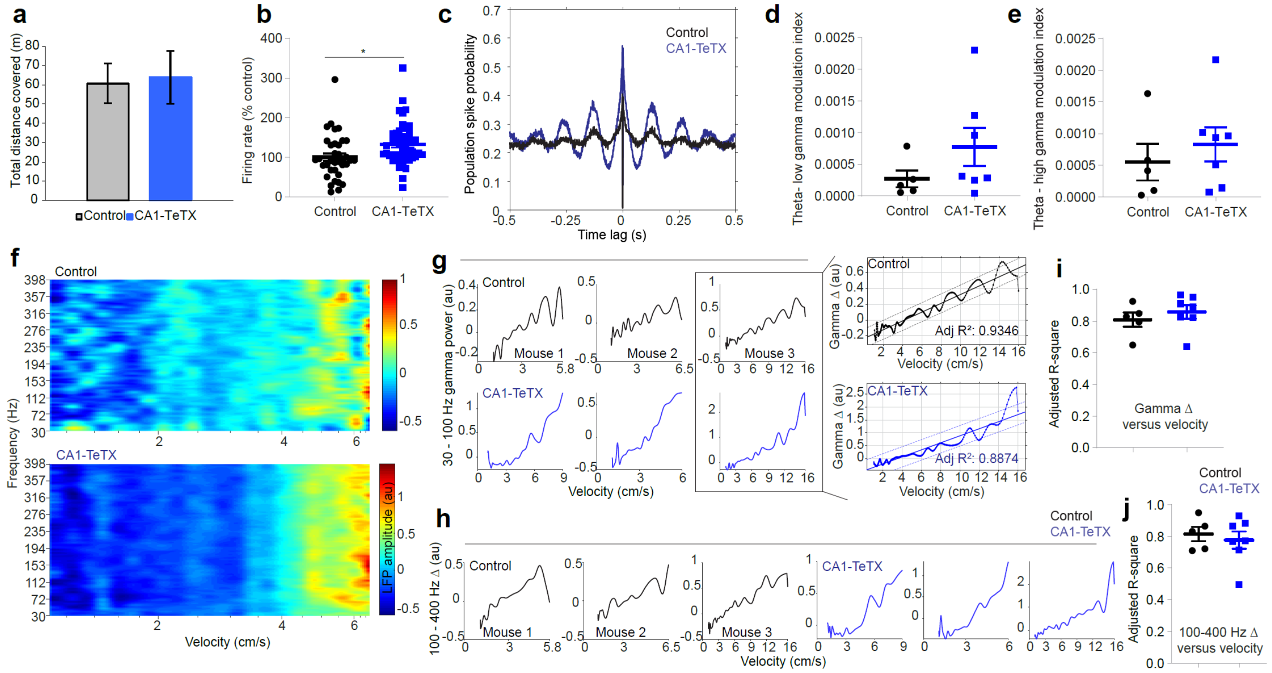

Supplementary Fig. 3: Impact of loss of neurotransmission in PCs on the theta or gamma during active exploration.

a Control and CA1-TeTX mice covered similar distances during active exploration.

b Mean spike rate during linear track exploration. Each data point represents a tetrode (unpaired two-sided t-test, T = 2.636, P = 0.010).

c Autocorrelogram of multiunit spikes during linear track exploration in control and CA1-TeTX mice.

d, e Theta phase and low (**d**) and high (**e**) gamma amplitude modulation index (Two-way RM ANOVA, freq. x genotype interaction, F (1, 10) = 1.144, P = 0.3099). N = 5 Control and 7 CA1-TeTX mice.

f Scalograms show the relationship between animal movement velocity and LFP power in control (top) and CA1-TeTX mice (bottom).

g, h Line plots show the relationship between the power of LFP gamma (**g**; 30 – 100 Hz) or LFP high-frequency (**h**; 100 - 400 Hz) and animal movement velocity from three representative mice in each group.

i, j Pearson R^2 between the amplitude of gamma or high-frequency and animal movement velocity. N = 5 Control and 7 CA1-TeTX mice.

Data shown in b, d, e represent mean ± s.e.m. * indicates P < 0.05.

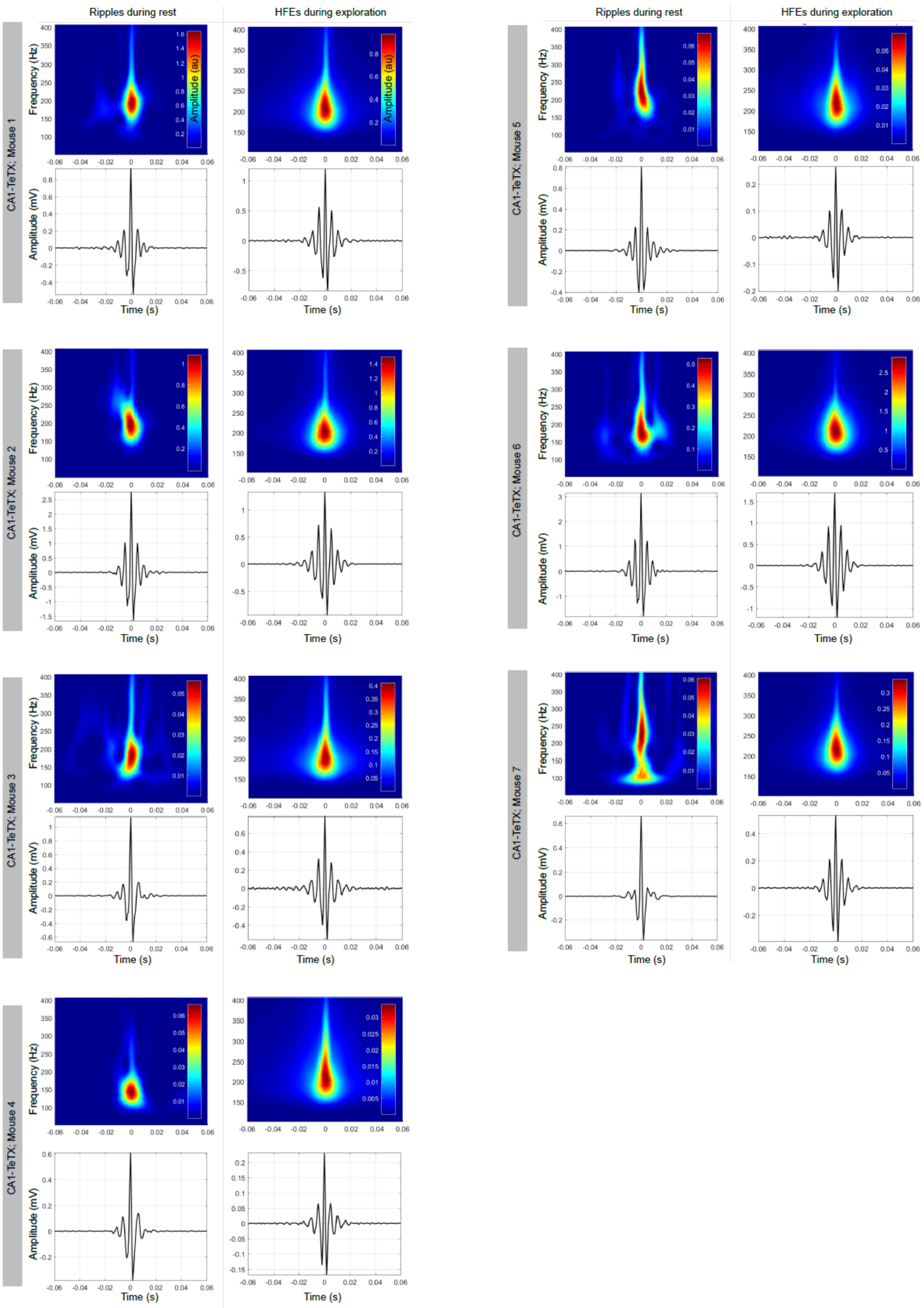

Supplementary Fig. 4: Waveform and magnitude scalogram of ripples and HFEs in CA1-TeTX mice. Mean magnitude scalograms and waveforms of ripples (during rest) and HFEs (during active exploration) in each of the 7 CA1-TeTX mice. au = arbitrary units.

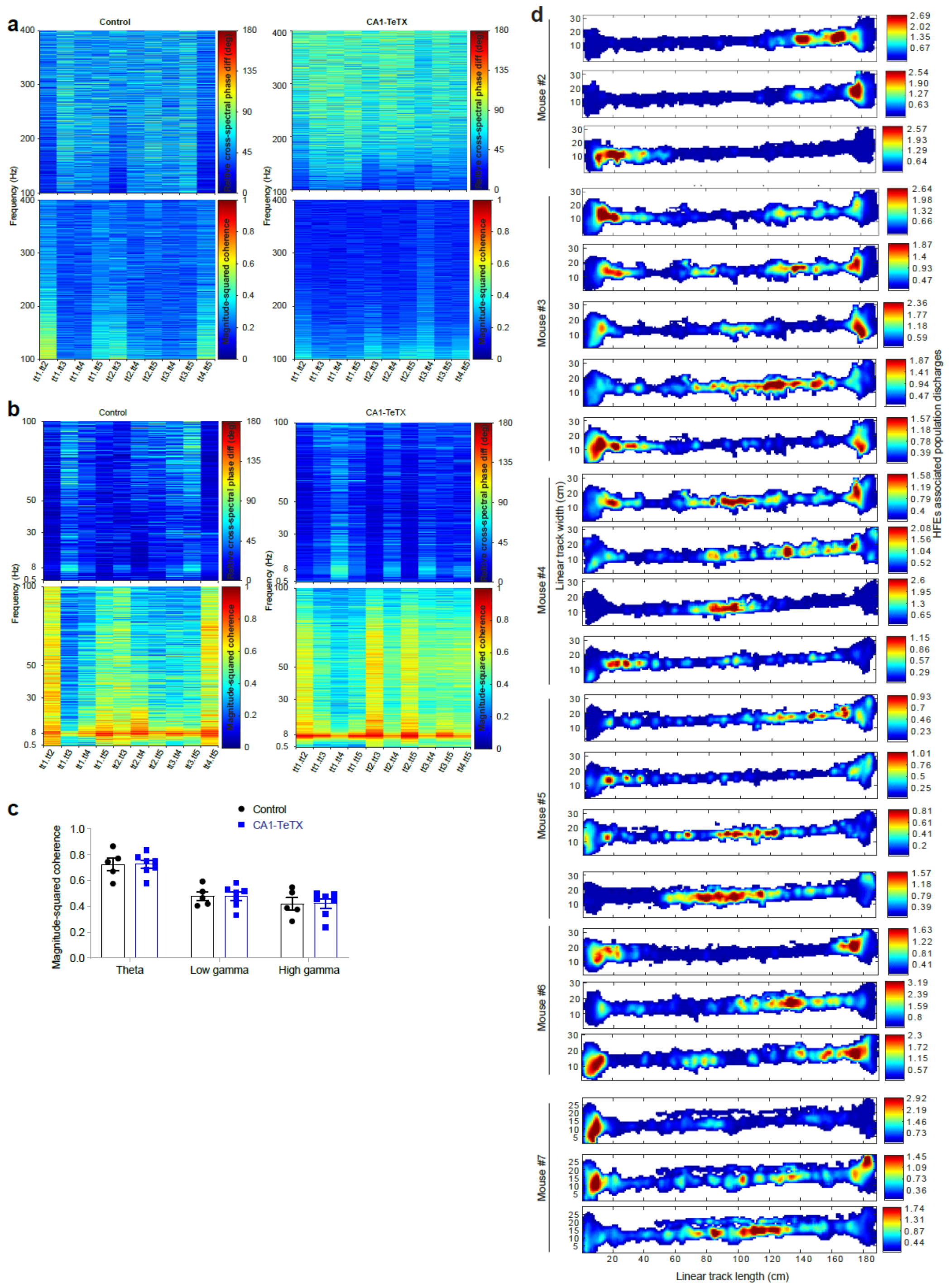

Supplementary Fig. 5: Place field-like maps of high-frequency events in CA1-TeTX mice.

a, b Plots show cross-spectral phase difference between pairs of tetrodes in CA1, and the corresponding magnitude squared coherence during active exploration sessions for high-frequency (**a**; 100 - 400 Hz) and 0.5 - 100 Hz (**b**) in control and CA1-TeTX mice.

c Bar plot shows the magnitude-squared coherence of theta (4 - 12 Hz; unpaired two-sided t-test, T = 0.0289, P = 0.977), low gamma (30 – 50 Hz; T = 0.016, P = 0.987), and high gamma (55 - 100 Hz; t = 0.0226, P = 0.982) between control and CA1-TeTX mice. N = 5 Control, & 7 CA1-TeTX mice. Data represent mean ± s.e.m.

d Place maps show the rate of HFEs time plotted by positional bins of the linear track. Each row corresponds to one CA1 location (tetrode). Tetrodes and the corresponding CA1-TeTX mice are indicated (note that CA1-TeTX mouse #1 is shown in Fig. 4k). All mice showed spatially selective HFE tetrodes, and across mice, we observed a total of 27 spatially selective tetrodes (TeTX1 = 5 tts (tetrodes), TeTX2 = 3 tts, TeTX3 = 5 tts, TeTX4 = 4 tts, TeTX5 = 4 tts, TeTX6 = 3 tts, TeTX7 = 3 tts) with one or more place field like patterns were observed per tetrode.

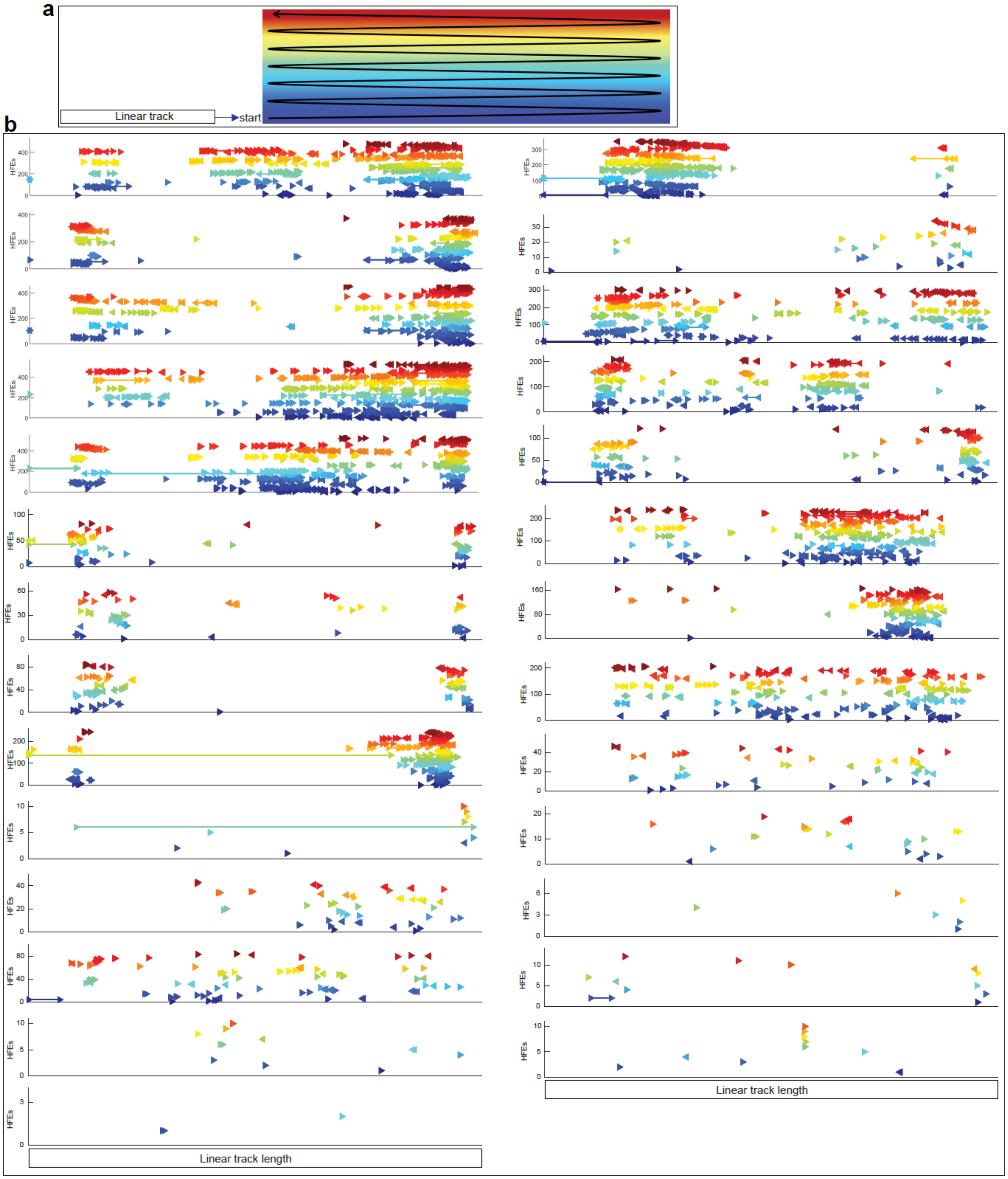

Supplementary Fig. 6: Place field-like maps of high-frequency events in CA1-TeTX mice.

a Experiment outline showing the linear track and the color coding for the early (blue) & late (red) laps.

b HFEs color-coded by the lap and order of occurrence within each session from dark blue (first) to dark red (last). Arrows indicate the direction of travel of the mouse (N = 7 CA1-TeTX mice).
