# Supplementary material for: Silencing CA1 pyramidal cells output reveals the role of feedback inhibition in hippocampal oscillations
Source: Nat Commun. 2024 Mar 11;15:2190. doi: 10.1038/s41467-024-46478-3 (PMC10928166; doi:10.1038/s41467-024-46478-3)
Supplement: Supplementary file 3 — Description of Additional Supplementary Files [file 41467_2024_46478_MOESM3_ESM.pdf]

Description of Additional Supplementary Files

Supplementary Data 1: A sample data for cumulative co-occurrence index (CCI) demo analysis. Five simultaneously recorded channels were used; each column represents an electrode. The numbers show indices of ripples occurring in that channel.
