# Supplementary material for: Silencing CA1 pyramidal cells output reveals the role of feedback inhibition in hippocampal oscillations
Source: Nat Commun. 2024 Mar 11;15:2190. doi: 10.1038/s41467-024-46478-3 (PMC10928166; doi:10.1038/s41467-024-46478-3)
Supplement: Supplementary file 5 — Reporting Summary [file 41467_2024_46478_MOESM5_ESM.pdf]

nature portfolio

Corresponding author(s): Thomas J. McHugh, Chinnakkaruppan Adaikkan

Last updated by author(s): Aug 26, 2023

Reporting Summary

Nature Portfolio wishes to improve the reproducibility of the work that we publish. This form provides structure for consistency and transparency in reporting. For further information on Nature Portfolio policies, see our Editorial Policies and the Editorial Policy Checklist.

Statistics

For all statistical analyses, confirm that the following items are present in the figure legend, table legend, main text, or Methods section.

n/a	Confirmed	
☐	☒	The exact sample size (*n*) for each experimental group/condition, given as a discrete number and unit of measurement
☐	☒	A statement on whether measurements were taken from distinct samples or whether the same sample was measured repeatedly
☐	☒	The statistical test(s) used AND whether they are one- or two-sided<br>*Only common tests should be described solely by name; describe more complex techniques in the Methods section.*
☐	☒	A description of all covariates tested
☐	☒	A description of any assumptions or corrections, such as tests of normality and adjustment for multiple comparisons
☐	☒	A full description of the statistical parameters including central tendency (e.g. means) or other basic estimates (e.g. regression coefficient) AND variation (e.g. standard deviation) or associated estimates of uncertainty (e.g. confidence intervals)
☐	☒	For null hypothesis testing, the test statistic (e.g. *F*, *t*, *r*) with confidence intervals, effect sizes, degrees of freedom and *P* value noted<br>*Give P values as exact values whenever suitable.*
☐	☒	For Bayesian analysis, information on the choice of priors and Markov chain Monte Carlo settings
☒	☐	For hierarchical and complex designs, identification of the appropriate level for tests and full reporting of outcomes
☐	☒	Estimates of effect sizes (e.g. Cohen's *d*, Pearson's *r*), indicating how they were calculated

Our web collection on statistics for biologists contains articles on many of the points above.

Software and code

Policy information about availability of computer code

Data collection	Zen acquisition program, Zeiss<br>pCLAMP 10, Molecular Devices, Version 10<br>Cheetah, Neuralynx, Version 5.7.4
Data analysis	GraphPad Prism , GraphPad , Version 8.3.1 & Version 9.3.1<br>ImageJ, NIH, Version 1.52a<br>pCLAMP10, Molecular Devices, Version 10<br>Matlab, MathWorks Version 2014a, 2019a, 2022b<br>MATLAB File Exchange: Circular Statistics Toolbox MATLAB File Exchange Function (https://www.mathworks.com/matlabcentral/fileexchange/10676-circular-statistics-toolbox-directional-statistics)<br>N/A<br>MATLAB File Exchange: Chronux Toolbox MATLAB File Exchange Function<br>(https://www.mathworks.com/matlabcentral/fileexchange/68537-chronux-analysis-software)<br>Version 2.12<br>SpikeSort3D, Neuralynx, Version 2.5.4<br>Neuraview, Neuralynx, Version 2.0.1<br>Matlab Import Export, Neuralynx, Version 6.0.0

For manuscripts utilizing custom algorithms or software that are central to the research but not yet described in published literature, software must be made available to editors and reviewers. We strongly encourage code deposition in a community repository (e.g. GitHub). See the Nature Portfolio guidelines for submitting code & software for further information.

Data

Policy information about availability of data

All manuscripts must include a data availability statement. This statement should provide the following information, where applicable:

- Accession codes, unique identifiers, or web links for publicly available datasets
- A description of any restrictions on data availability
- For clinical datasets or third party data, please ensure that the statement adheres to our policy

This study did not generate new unique reagents. All data necessary to assess the conclusions of this research are available in the text and supplementary materials. Any additional information required to reanalyze the data reported in this paper is available from the lead contact upon request.

Research involving human participants, their data, or biological material

Policy information about studies with human participants or human data. See also policy information about sex, gender (identity/presentation), and sexual orientation and race, ethnicity and racism.

Reporting on sex and gender	*Use the terms sex (biological attribute) and gender (shaped by social and cultural circumstances) carefully in order to avoid confusing both terms. Indicate if findings apply to only one sex or gender; describe whether sex and gender were considered in study design; whether sex and/or gender was determined based on self-reporting or assigned and methods used. Provide in the source data disaggregated sex and gender data, where this information has been collected, and if consent has been obtained for sharing of individual-level data; provide overall numbers in this Reporting Summary. Please state if this information has not been collected. Report sex- and gender-based analyses where performed, justify reasons for lack of sex- and gender-based analysis.*
Reporting on race, ethnicity, or other socially relevant groupings	*Please specify the socially constructed or socially relevant categorization variable(s) used in your manuscript and explain why they were used. Please note that such variables should not be used as proxies for other socially constructed/relevant variables (for example, race or ethnicity should not be used as a proxy for socioeconomic status). Provide clear definitions of the relevant terms used, how they were provided (by the participants/respondents, the researchers, or third parties), and the method(s) used to classify people into the different categories (e.g. self-report, census or administrative data, social media data, etc.) Please provide details about how you controlled for confounding variables in your analyses.*
Population characteristics	*Describe the covariate-relevant population characteristics of the human research participants (e.g. age, genotypic information, past and current diagnosis and treatment categories). If you filled out the behavioural & social sciences study design questions and have nothing to add here, write "See above."*
Recruitment	*Describe how participants were recruited. Outline any potential self-selection bias or other biases that may be present and how these are likely to impact results.*
Ethics oversight	*Identify the organization(s) that approved the study protocol.*

Note that full information on the approval of the study protocol must also be provided in the manuscript.

Field-specific reporting

Please select the one below that is the best fit for your research. If you are not sure, read the appropriate sections before making your selection.

☒ Life sciences ☐ Behavioural & social sciences ☐ Ecological, evolutionary & environmental sciences

For a reference copy of the document with all sections, see nature.com/documents/nr-reporting-summary-flat.pdf

Life sciences study design

All studies must disclose on these points even when the disclosure is negative.

Sample size	Sample size were estimated based on previous studies.
Data exclusions	No data was excluded after acquisition & or analysis
Replication	Ex vivo slice experiments; there was no day or slice effect- results were reproducible across days and slices. In vivo experiments; there were no litter-mate/batch effects - results were reproducible throughout the duration of the project. For both ex vivo and in vivo experiments, multiple batches of AAVs were used with all producing similar effects (reporter expression & physiological effects)- no batch effect was observed.
Randomization	In all experiments, litter-mate mice were split into multiple groups. Mice were age and gender matched across all experiments.
Blinding	In vivo electro-physiological data analysis was done experimenters blinded to the genotypes.

Reporting for specific materials, systems and methods

We require information from authors about some types of materials, experimental systems and methods used in many studies. Here, indicate whether each material, system or method listed is relevant to your study. If you are not sure if a list item applies to your research, read the appropriate section before selecting a response.

Materials & experimental systems

n/a	Involved in the study
☐	☒ Antibodies
☒	☐ Eukaryotic cell lines
☒	☐ Palaeontology and archaeology
☐	☒ Animals and other organisms
☒	☐ Clinical data
☒	☐ Dual use research of concern
☒	☐ Plants

Methods

n/a	Involved in the study
☒	☐ ChIP-seq
☒	☐ Flow cytometry
☒	☐ MRI-based neuroimaging

Antibodies

Antibodies used

Antibodies (dilutions) Catalogue number RESOURCE SOURCE IDENTIFIER
Anti-GAD67 (1:500) Sigma-Aldrich Cat#MAB5406; RRID:AB_2278725
Anti-Neun (1:1000) Synaptic Systems Cat#266 004; RRID:AB_2619988
Anti-Synaptobrevin 2 (1:250) Synaptic Systems Cat#104 202; RRID:AB_887810
Donkey anti-Mouse, Alexa Fluor 488 Invitrogen Cat# A21202 Thermo Fisher Scientific Cat# A21202; AB_141607
Anti-Guinea Pig IgG (H+L) Secondary Antibody, Alexa Fluor® 647 conjugate (1:1000) Thermo Fisher Scientific Cat#A-21450 21450; RRID:AB_141882
Anti-Rabbit IgG (H+L) Secondary Antibody, Alexa Fluor® 647 conjugate (1:1000) Thermo Fisher Scientific Cat#A-31573; RRID:AB_2536183

Validation

All anitbodies used were validated by the commercial supplier(s) mentioned above

Animals and other research organisms

Policy information about studies involving animals; ARRIVE guidelines recommended for reporting animal research, and Sex and Gender in Research

Laboratory animals

CaMKIIα::Cre; JAX#005359, mice, C57BL/6J

Wild animals

N/A

Reporting on sex

Male and female animals were used for all experiments

Field-collected samples

N/A

Ethics oversight

Approved by RIKEN Animal Care and Use Committee, & the committee for animal care of the Division of Comparative Medicine (DVM) at the Massachusetts Institute of Technology (MIT).

Note that full information on the approval of the study protocol must also be provided in the manuscript.
